# Dynamics of exciton polaron in microtubule

**DOI:** 10.1016/j.heliyon.2022.e08897

**Published:** 2022-02-02

**Authors:** W.A. Nganfo, C. Kenfack-Sadem, A.J. Fotué, M.C. Ekosso, S.N. Wopunghwo, L.C. Fai

**Affiliations:** Condensed Matter and Nanomaterials, Faculty of Science, Department of Physics, University of Dschang, Po Box 67, Cameroon

**Keywords:** Microtubule, Exciton-polaron, Anisotropic, Vibration frequency, Protofilament, Helix, Antihelix

## Abstract

In this paper, we study the dynamical properties of the exciton-polaron in the microtubule. The study was carried out using a unitary transformation and an approximate diagonalization technique. Analytically, the modeling of exciton-polaron dynamics in microtubules is presented. From this model, the ground state energy, mobility, and entropy of the exciton-polaron are derived as a function of microtubule's parameters. Numerical results show that, depending on the three vibrational modes (protofilament, helix, antihelix) in MTs, exciton-polaron energy is anisotropic and is more present on the protofilament than the helix and absent on the antihelix. Taking into account the variation of the protofilament vibrations by fixing the helix vibrations, exciton-polaron moves between the 1st and 2nd protofilaments. It is seen that the variation of the two vibrations induces mobility of the quasiparticle between the 1st and 15th protofilament. This result points out the importance of helix vibrations on the dynamics of quasiparticles. It is observed that the mobility of the exciton polaron and the entropy of the system are strongly influenced by the vibrations through the protofilament and helix. The effects of the one through the antihelix is negligible. The entropy of the system is similar to that of mobility. Confirming that the quasiparticles move in the protofilament faster than in the helix.

## 1 Introduction

**Figure 1:**
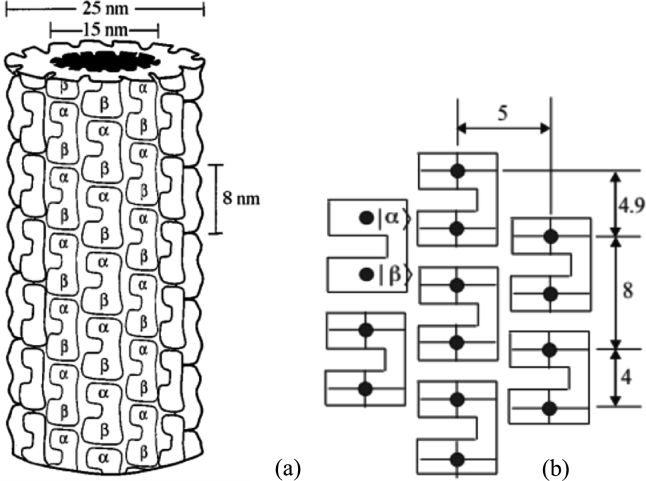
(a) Model of the microtubule made up of 13 protofilaments and formed from *αβ* tubulin dimers. (b) Each filled circle represents a possible location of the electron with a corresponding quantum state |*α*〉 (upper) or 〈*β*| (lower) (Hiramatsu et al., 2008).

Microtubules (MTs), major elements of the cytoskeleton are alleged to be at the middle of cellular organization and knowledge processing. They play a crucial role in intracellular transport where they function as road-rail for motor proteins, essential during cellular division and cell motility (Horio and Murata, 2014; Ganguly et al., 2012). An MT is a long cylindrical tube of about 25 nm in outer diameter and 14 nm in inner diameter. The interior of the cylinder is likely to be filled with ordered water molecules, which implies the existence of electric dipoles and electric fields. (Wang et al., 2012; Rüdiger et al., 2016; Amos and Klug, 1974). Formed by thirteen protofilaments, MTs are extremely dynamic and unstable due to the dynamic behavior of their basic units called tubulin dimers that attach end to end to make a protofilament (Antal et al., 2007; Aher and Akhmanova, 2018). Each tubulin dimer consists of two elements, *α* tubulin negatively charged and *β* tubulin positively charged. This polarity difference implies that MTs are polarised structures where tubulin dimers are seen as electric dipoles (Stracke et al., 2002; Pokorný, 2004). The *αβ* tubulins are represented within the protofilament as double-well potential, where the mobile electron on each *αβ* tubulin dimer is often localized within the site of the monomer *α* or the location of the monomer *β* (Hiramatsu et al., 2008; Ying et al., 2004; Mavromatos et al., 2002). Under certain conditions, and by quantum tunneling phenomenon, an electron may go from one well into another (Chen et al., 2005). Thus, depending on the situation of the mobile electron, the tubulin dimer has two basic states as shown in Fig. 1.

When the electrons present within the double-well potential is excited, it crosses from the fundamental state to the excited state, leaving a hole. This interaction between electron and hole forms an exciton. Several authors have studied the properties of the exciton in microtubules (Tuszyński et al., 1998). Portet et al. (2005) studied the exciton energy transfers between MT dipoles and showed that the exciton energy is between 64-68 meV. Celardo et al. (2019) found that the spreading of excitation is ballistic in the absence of external sources of disorder and strongly hooked into initial conditions in MT. Kurian et al. (2017) gave the evolution of exciton states in tubulin.

Microtubules are protein aggregates that depolymerize due to GTP hydrolysis. The energy produced during hydrolysis creates phonon vibrations in the MT network. By considering them as an aggregated layer in two dimensions, several authors have studied the lattice vibrations of MTs. Pokorný (1999) suggested that vibrations are possible in microtubules; tubulin dimers being electric dipoles, their vibrations generate an electrical field. Pokorný (2004) studied the excitation of vibrations in microtubules in living cells. Thackston et al. (2019) measured the electrical field produced by vibrations of the microtubule lattice and showed that a vibrating MT exerts significant forces on electric dipoles. There are several other articles that handle the vibrational properties of MTs (Shirmovsky and Shulga, 2018; Taj and Zhang, 2012). The elastic vibrations of the tubulins around their equilibrium position within the network are called phonons (Sirenko et al., 1996a, Sirenko et al., 1996b; Stroscio and Dutta, 2001). Sirenko et al., 1996a, Sirenko et al., 1996b studied several vibrational modes in MTs (radial, torsional and longitudinal) and showed that the velocity of the phonons varies between 200 and 600 m/s. Pokorný (2004) constructed a model of MT as monoatomic linear chains and concluded that the expected phonon vibration frequencies were of the order of 10^7^-10^10^ m﹨s. Portet et al. (2005) expressed the frequencies and vibration speed of the phonon as a function of the elastic constants characterizing the geometry of the MTs. They said that there are three modes of vibration in microtubules: The one following the protofilament, the one following helix, and the one following antihelix. The choices of elasticity constancies are justified by the fact that Sept et al. (2003), Kis et al. (2002), and Nogales et al. (1999) reported that tubulin dimers are strongly bound along the protofilament, while the interaction between protofilaments is much weaker. So they considered that $k_{p}≻k_{h},k_{a}$. Li et al. (2002) specified that the longitudinal intra-protofilament interactions are identical while the lateral interactions between the protofilaments are different therefore $k_{h}$ is different from $k_{a}$. In the work of Sept et al. (2003), they estimated that the elasticity constant corresponding to the harmonic approximation is k=4N/m. de Pablo et al. (2003) experimentally estimated that the elasticity constant linked to the lateral interactions between protofilaments is k=0.1N/m. Portet et al. (2005) suggest that the elastic constancies are anisotropic and can vary following the directions, in their studies, they considered that $k_{p}=4.5N/m$, $k_{h}=0.1N/m$, $k_{h}=0.01N/m$ and the mass of the dimer is taken to be $m=1.89\times 10^{-22}kg$. Several authors (Cifra et al., 2011; Thackston et al., 2019) have shown that microtubules have an intrinsic increasing electric field resulting from network vibrations and an external electric field resulting from vibrations of other components of the cytoskeleton. Pokorný (2004) and Pokorný et al. (2005) showed that endogenous electric fields by action on charges and by polarization exert forces that can lead to charges and particles in the cell. They analyzed the mobility of mass and electrons driven by deterministic forces with the latter by thermal forces.

The electron-hole-phonon coupling also known as the exciton-phonon interaction is understood within the microtubule literature and mentioned by (Kurian et al., 2017; Craddock et al., 2014; Craddock and Tuszynski, 2010). Also, it is well known that the electron-phonon interaction creates the quasi-particle called polaron. So, the exciton-polaron appears when the exciton interacts with optical or acoustic phonons. Radoń et al. (2022), studied ultraslow electron-phonon scattering and polaron formation in magnetite to find relations between the formation of polarons, phonons, and conduction by a virtual free electron gas. The analysis performed here shows that the interaction electron-phonon coupling results in the formation of large polarons, and these are responsible for high-frequency conductivity in magnetite. Chorošajev et al. (2014) studied the dynamics of the formation of polaron excitons in molecular systems coupled to an overamplified bath using the Dirac-Frenkel variational principle and the Davydov Ansatz D1. They showed that the timescale of the polaron formation can be defined by the timescale of resonant coupling quenching. Kato et al. (1999) studied excitonic polarons in molecular aggregates: by performing a dynamic coherent potential approximation. In their study, excitons interact with phonons to form exciton-polaron in molecular aggregates. Tuszyński et al. (1999), studied the mechanisms of exciton energy transfer in protein aggregates. In particular, they addressed the issue of determining the strength of the exciton-phonon interaction and its effect on the formation and dynamics of a coherent exciton domain. They specify that for certain coupling values there is the formation of polaron in the protein aggregates. Craddock and Tuszynski (2010) studied a critical assessment of the information processing capabilities of neuronal microtubules using coherent excitations. They showed that phonons, excitons, and polarons exist in microtubules. A phase diagram is constructed in terms of the parameter space spanned by the coefficient *γ* which represents the ratio of the phonon energy to the exciton energy and the coefficient g which is one half of the product of the square of the ratio of the exciton–phonon energy to the dipole-dipole energy times the ratio of the exciton energy to the phonon energy. It is straightforward to estimate the range of the coefficient values as 0.03 < *γ* < 1.3 and 0 < g < 0.001. This falls squarely into region III, i.e., a small polaron system. Most importantly, the radiative decay of the small polaron domain at 300 K is expected not to exceed 10^−10^ s.

Our interest in the exciton-polaron concept comes from the fact that many studies have proven that the behavior of an exciton is constantly modified by its interaction strength with the phonon (Thilagam, 2015; Chen, 2018). The latter is therefore important for understanding the conductivity within materials. Exciton-polarons present a higher degree of lattice deformation than the conventional polaron. The above-mentioned characteristics prove the contribution of the exciton-polaron to the dynamics of a given system. This concept is well known in the context of nanostructure dynamics (Mommadi et al., 2020), but to our knowledge, it has not yet been studied in the context of microtubule dynamics. MTs consist of dynamically aggregated proteins that move with the phenomena of polymerization and depolymerization, maintaining the mechanical stability of the cell. During these processes, several physical and biological transformations take place in and around microtubules (Gudimchuk and McIntosh, 2021; Borin et al., 2020). In the above works, it is clearly demonstrated that there is the formation of excitons, phonons, electrons, and polarons in MTs. For this reason, in the present work, we investigate the dynamics of exciton-polaron quasi particles on the dynamics of microtubules. The effects of microtubule parameters on exciton-polaron dynamics will also be studied. The variational method will be used to evaluate the ground state's energy, mobility, and entropy of the exciton-polaron according to the parameters characterizing the geometry of the microtubules. The paper is organized as follows: Section 2 presents the model and the calculations. The results are discussed in section 3, and section 4 concludes.

## 2 Model and calculations

In this section, the conceptualization of exciton, phonon, exciton-phonon interaction, and exciton-polaron will be presented in microtubules using the basic assumptions. Then we use the unitary transformation presented by (Thilagam, 2015; Thilagam and Singh, 1996), to diagonalize as much as possible the exciton-phonon interaction operators because the Hamiltonian consisting of the exciton, phonon, and exciton-phonon interaction energy operators is not diagonal. Finally, we will use the variational method to evaluate the ground state energy of the exciton-polaron.

### 2.1 Model

We used the double-well potential to represent each dimer consisting of the *αβ*-tubulins in the protofilament. The exciton present in the double-well arises from the electron-hole interaction when the coulomb interaction force is strong in the dimers which are electric dipoles. The exciton is localised in the XY plane and thus its motion becomes two-dimensional. The energy from hydrolysis creates a quantum of vibrations which are phonons. The polaron comes from the electron phonon interaction. Then electrons, hole, phonon interaction create the quasi-particles called exciton-polaron. For clarity, we will denote all wave vectors in the plane by the index *l*. The Hamiltonian of the exciton-polaron is given by the following equation:

(1) $$\overset{ˆ}{H}={\overset{ˆ}{H}}_{ex}^{Q2D}+{\overset{ˆ}{H}}_{ph}+{\overset{ˆ}{H}}_{ex-ph}^{Q2D}$$

The first term ${\overset{ˆ}{H}}_{ex}^{Q2D}$ represents the Hamiltonian of an exciton evolving in two dimensions, the second term ${\overset{ˆ}{H}}_{ph}$ represents the energy of the phonons and the last term ${\overset{ˆ}{H}}_{ex-ph}^{Q2D}$ gives the interaction energy of exciton-phonon. Q2D denotes the interaction between the quasi-two-dimensional exciton and phonons in double-well. The corresponding expression of each term is given as follows:

(2) $${\overset{ˆ}{H}}_{ex}^{Q2D}=\sum_{k_{l},z}E^{ex}(k_{l})a_{K}^{+}a_{K}+H_{Z}.$$

Assuming that all the wave vectors of the plane are indexed by *l*. Where $E^{ex}\left(k_{l}\right)$ is the energy of the exciton in a double-well, $a_{K}^{+}$ and $a_{K}$ are respectively the creation and annihilation operators of an exciton. The term $E^{ex}$ is expressed as:

(3) $$E^{ex}=\hbar \Omega +2\sum_{i=1}^{N}\sum_{j=1}^{3i}J_{ij}\cos\left(\overset{\to }{k}.\overset{\to }{r_{ij}}\right)+\frac{\hbar^{2}k_{l}^{2}}{2M^{⁎}}$$

The term $\hbar \Omega +2\sum_{i=1}^{N}\sum_{j=1}^{3i}J_{ij}\cos\left(\overset{\to }{k}.\overset{\to }{r_{ij}}\right)$ in Eq. (3) is the dispersion energy of exciton in the MT and *ħ*Ω defines the energy difference between the ground and first excited state (Craddock et al., 2014). *i* and *j* determine the neighborhood dimers, $J_{ij}$ is the dipole-dipole interaction energy of the central dimer dipole and a given neighboring dipole. $r_{ij}$ is the distance between two dipoles, $k_{l}$ is the wave vector of the exciton in the XY plane and $M^{⁎}=m_{e}^{⁎}+m_{h}^{⁎}$ is the effective mass of the exciton. $m_{e}^{⁎}$ is the mass of electron; $m_{h}^{⁎}$ is the mass of the hole. The Hamiltonian $H_{Z}$ of the exciton in the z-direction is given by:

(4) $$H_{z}=\frac{p_{e,z}^{2}}{2m_{e}^{⁎}}+\frac{p_{h,z}^{2}}{2m_{h}^{⁎}}+V_{e}\left(z_{e}\right)+V_{h}(z_{h})$$

Where $p_{e,z}$ and $p_{h,z}$ are the momentum components associated respectively with electrons ($z_{e}$) and hole ($z_{h}$). $v_{e}\left(z_{e}\right)$ and $v_{h}\left(z_{h}\right)$ are respectively the confinement potentials of the electron and hole between interlayer of the dimer system.

The Hamiltonian of phonon ${\overset{ˆ}{H}}_{ph}$ is given by:

(5) $$H_{ph}=\sum_{q}\hbar \omega_{p,h}\left(b_{q}^{+}b_{q}+\frac{1}{2}\right)$$

In Eq. (5), the terms $b_{q}^{+}$($b_{q}$) denote the creation (annihilation) operators of phonons. *q* is the wave vector of the phonon, $\hbar \omega_{p,h}$ is the phonon energy, $\omega_{p,h}$ represents the vibration frequency following the protofilament *p* and helix *h* (Craddock and Tuszynski, 2010).

The Hamiltonian of the quasi-two-dimensional exciton-phonon interaction is given by:

(6) $${\overset{ˆ}{H}}_{ex-ph}^{Q2D}=\sum_{k_{l},q_{l,}}\chi_{eff}\left(b_{q_{l}}+b_{-q_{l}}^{+}\right)a_{k_{l}+q_{l}}^{+}a_{k_{l}}$$

Where

(7) $$\chi_{eff}=2J\alpha \sqrt{M_{D}K_{B}T_{ph}}d$$

$M_{D}$ is the mass of tubulin; $K_{B}$ is the Boltzmann's constant; *d* is the distance between the dimers, *J* is the dipole-dipole interaction (Tuszyński et al., 1999). By substituting Eqs. (2), (5) and (6) into Eq. (1), the final expression of the Hamiltonian of exciton polaron in MT is written as:

(8) $$\overset{ˆ}{H}=\sum_{k_{l},z}E^{ex}(k_{l},z)a_{k_{l}}^{+}a_{k_{l}}+\frac{p_{e,z}^{2}}{2m_{e}^{⁎}}+\frac{p_{h,z}^{2}}{2m_{h}^{⁎}}+\sum_{q}\hbar \omega_{q}\left(b_{q}^{+}b_{q}+\frac{1}{2}\right)+\sum_{k_{l},q_{l,}}\chi_{eff}\left(b_{q_{l}}+b_{-q_{l}}^{+}\right)a_{k_{l}+q_{l}}^{+}a_{k_{l}}$$

### 2.2 Approximate diagonalization method

Our aim is to compute the ground state energy of the exciton-polaron in an MT. So, to achieve that, we have to use the approximate diagonalization method (Thilagam, 2015; Thilagam and Singh, 1996) on Hamiltonian given in Eq. (8). First of all, we partially use the unitary transformation $U_{ex}=e^{is}$ proposed by Singh (Singh, 2013, Singh, 1994).

With

(9) $$S=\sum_{k_{l},q_{l}}a_{\left(k_{l}+q_{l}\right)}^{+}a_{k_{l}}\left[f_{ex}^{⁎}(k_{l},q_{l})b_{-q}^{+}+f_{ex}(k_{l},q_{l})b_{q}\right]$$

Using the series expansion of the transformed Hamiltonian $U_{ex}^{-1}\overset{ˆ}{H}U_{ex}$ (Singh, 2013) the expression of the Diagonalization is written as:

(10) $$U_{ex}^{-1}\overset{ˆ}{H}U_{ex}=U_{ex}^{-1}{\overset{ˆ}{H}}_{0}U_{ex}+U_{ex}^{-1}{\overset{ˆ}{H}}_{ex-ph}^{2D}U_{ex}$$

Eq. (10) allows finding the form of the function $f_{ex}(k_{l},q_{l})$ given in Eq. (9).

The use of the Baker-Campbell-Hausdorff formula (Oteo, 1991) given by:

(11) $$e^{-i\overset{ˆ}{A}\lambda }\overset{ˆ}{B}e^{i\overset{ˆ}{A}\lambda }=\overset{ˆ}{B}-i\lambda \left[\overset{ˆ}{A},\overset{ˆ}{B}\right]+\frac{\lambda }{2!}\left[i\overset{ˆ}{A},i\left[\overset{ˆ}{A},\overset{ˆ}{B}\right]\right]+...,$$

Applying this formula into Eq. (10), we obtain the following series expansion:

(12) $$U_{ex}^{-1}\overset{ˆ}{H}U_{ex}={\overset{ˆ}{H}}_{0}+\left(i{\left[{\overset{ˆ}{H}}_{0},S\right]}_{-}+{\overset{ˆ}{H}}_{ex-ph}^{2D}\right)+i\left({\left[\frac{i}{2}{\left[{\overset{ˆ}{H}}_{0},S\right]}_{-}+{\overset{ˆ}{H}}_{ex-ph}^{2D},S\right]}_{-}\right)+...$$

In order 2, the perturbation theory allows us to write:

(13) $$\left[{\overset{ˆ}{H}}_{0},S\right]={\overset{ˆ}{H}}_{ex-ph}^{2D}$$

Since:

(14) $$\left[{\overset{ˆ}{H}}_{0},S\right]=[\sum_{k_{l},z}E^{ex}(k_{l},z)a_{k}^{+}a_{k}+H_{Z}+\sum_{q}\hbar \omega b_{q}^{+}b_{q},\sum_{k_{l},q_{l}}a_{\left(k_{l}+q_{l}\right)}^{+}a_{k_{l}}\left(f_{ex}^{⁎}(k_{l},q_{l})b_{-q}^{+}+f_{ex}(k_{l},q_{l})b_{q}\right)]$$

Using the following commutator relationships:

(15) $$\left[a_{j},a_{i}^{+}\right]=\left(1\right)\delta_{ij}\;\left[a_{i}^{+},a_{j}\right]=\left(-1\right)\delta_{ij}$$

Eq. (13) becomes:

(16) $${\overset{ˆ}{H}}_{ex-ph}^{2D}=\sum_{k_{l},q_{l}}a_{\left(k_{l}+q_{l}\right)}^{+}a_{k_{l}}f_{ex}^{⁎}b_{-q_{l}}^{+}\left(E_{\left(k_{l}+q_{l}\right)}^{ex}-E_{k_{l}}^{ex}+\hbar \omega \right)++\sum_{k_{l},q_{l}}a_{\left(k_{l}+q_{l}\right)}^{+}a_{k_{l}}f_{ex}b_{q_{l}}\left(E_{\left(k_{l}+q_{l}\right)}^{ex}-E_{k_{l}}^{ex}-\hbar \omega \right)=\sum_{k_{l},q_{l,}}\chi_{eff}\left(b_{q_{l}}+b_{-q_{l}}^{+}\right)a_{k_{l}+q_{l}}^{+}a_{k_{l}}$$

By comparing both sides of Eq. (16), the following expressions of $f_{ex}$

(17) $$f_{ex}^{⁎}=\frac{\chi_{eff}}{E_{\left(k_{l}+q_{l}\right)}^{ex}-E_{\left(k_{l}\right)}^{ex}+\hbar \omega }\;\mathrm{and}f_{ex}=\frac{\chi_{eff}}{E_{\left(k_{l}+q_{l}\right)}^{ex}-E_{\left(k_{l}\right)}^{ex}-\hbar \omega }$$

It is noted that the transformation of the Hamiltonian as applied above is only interesting when the functions derived from $f_{ex}^{⁎}$ and $f_{ex}$ are small, otherwise, the series $U_{ex}^{-1}{\overset{ˆ}{H}}_{ex}U_{ex}={\overset{ˆ}{H}}_{0}+[i\{{\overset{ˆ}{H}}_{0},S\}.\pm {\overset{ˆ}{H}}_{ex-ph}^{2D}+i\{\frac{1}{2}i\ldots$ does not converge. So let us diagonalize the matrix to find its diagonal shape, the transformed Hamiltonian $H^{T}=U_{ex}^{-1}\overset{ˆ}{H}U_{ex}$ is obtained by diagonalizing each term of *H*.

The final expression of the transformed Hamiltonian is given by:

(18) $${\overset{ˆ}{H}}^{T}={\overset{ˆ}{H}}_{ex}^{Q2D}-i\sum_{k_{l},q_{l}}\sum_{k_{l}^{\prime },q_{l}^{\prime }}E_{k_{l}}^{ex}a_{\left(k_{l}^{\prime }+q_{l}\right)}^{+}a_{k_{l}^{\prime }}(f_{ex}^{⁎}b_{-q_{l}}^{+}+f_{ex}b_{q_{l}})+{\overset{ˆ}{H}}_{ph}+{\overset{ˆ}{H}}_{z}+{\overset{ˆ}{H}}_{ex-ph}^{2D}+i\sum_{k_{l}}\sum_{k_{l}^{\prime },q_{l}}\chi_{eff}\left(f_{ex}^{⁎}-f_{ex}\right)a_{\left(k_{l}^{\prime }+q_{l}\right)}^{+}a_{k_{l}}a_{\left(k_{l}^{\prime }+q_{l}\right)}^{+}a_{k_{l}^{\prime }}$$

Eq. (18) can also be written as:

(19) $${\overset{ˆ}{H}}^{T}={\overset{ˆ}{H}}_{ex}^{Q2D}+{\overset{ˆ}{H}}_{ph}+{\overset{ˆ}{H}}_{z}+{\overset{ˆ}{H}}_{ex-ph}^{\prime \;T2D}+{\overset{ˆ}{H}}^{\prime }$$

where

(20) $${\overset{ˆ}{H}}_{ex-ph}^{\prime \;T2D}=i\sum_{k_{l}}\sum_{k_{l}^{\prime },q_{l}}\chi_{eff}\left(f_{ex}^{⁎}-f_{ex}\right)a_{\left(k_{l}^{\prime }+q_{l}\right)}^{+}a_{k_{l}}a_{\left(k_{l}^{\prime }+q_{l}\right)}^{+}a_{k_{l}^{\prime }}$$

and

(21) $${\overset{ˆ}{H}}^{\prime }={\overset{ˆ}{H}}_{ex-ph}^{2D}-i\sum_{k_{l},q_{l}}\sum_{k_{l}^{\prime },q_{l}^{\prime }}E_{k_{l}}^{ex}a_{\left(k_{l}^{\prime }+q_{l}\right)}^{+}a_{k_{l}^{\prime }}(f_{ex}^{⁎}b_{-q_{l}}^{+}+f_{ex}b_{q_{l}})$$

${\overset{ˆ}{H}}^{\prime }$ represents a part of the interaction operator that cannot be diagonalized by the above transformation. Its contribution can be expected to be neglected. The total Hamiltonian is not completely diagonal, this is why it is an approximation. This approximation provides an analytical way to estimate the energy of the exciton-polaron. So, Eq (19) is rewritten as:

(22) $${\overset{ˆ}{H}}^{T}\approx {\overset{ˆ}{H}}_{ex}^{Q2D}+{\overset{ˆ}{H}}_{ph}+{\overset{ˆ}{H}}_{z}+{\overset{ˆ}{H}}_{ex-ph}^{\prime \;T2D}$$

### 2.3 The exciton-polaron energy in the microtubule

The ground state energy of exciton-polaron is computed using the state vector $|k_{l},n(q)\rangle$, as:

(23) $$|k_{l},n(q)\rangle =a_{k_{l}}^{+}|0,n(q_{l},q_{z})\rangle$$

With $|0;n(q_{l},q_{z})\rangle =|0\rangle |n(q_{l},q_{z})\rangle$, |0〉 denotes the vacuum state vector of exciton and $|n(q_{l})\rangle =|n_{q_{1}},n_{q_{2}},n_{q_{3}}...\rangle$ the bulk phonon state vector with n(q) being the occupation number of phonons with wave vector $n(q_{l},q_{z})$. For the ground state of an exciton-polaron, n(q)=0, then the ground-state energy can be evaluated using:

(24) $$E_{pol}^{ex}(0)=\langle n(q),k_{l}|U_{ex}^{-1}{\overset{ˆ}{H}}^{T}U_{ex}|k_{l},n(q)\rangle$$

Performing calculations, the ground state energy is obtained as:

(25) $$E_{pol}^{ex}(0)=E_{k_{l}}^{ex}+\hbar \omega +E_{z}^{ex}-\sum_{k_{l},q_{l}}{\left|\chi_{eff}\right|}^{2}\times \left[\frac{1}{E_{\left(k_{l}+q_{l}\right)}^{ex}-E_{\left(k\right)}^{ex}-\hbar \omega }-\frac{1}{E_{\left(k_{l}+q_{l}\right)}^{ex}-E_{\left(k\right)}^{ex}+\hbar \omega }\right]$$

In Eq. (25), the summation term has to be estimated. It is given by:

(26) $$I_{ex}(K)=\sum_{k_{l},q_{l}}{\left|\chi_{eff}\right|}^{2}\times \left[\frac{1}{E_{\left(k_{l}+q_{l}\right)}^{ex}-E_{\left(k\right)}^{ex}-\hbar \omega }-\frac{1}{E_{\left(k_{l}+q_{l}\right)}^{ex}-E_{\left(k\right)}^{ex}+\hbar \omega }\right]$$

Where

(27) $$E_{k_{l}}^{ex}=\hbar \Omega +2\sum_{i=1}^{N}\sum_{j=1}^{3i}J_{ij}\cos\left(\overset{\to }{k}.\overset{\to }{r_{ij}}\right)+\frac{\hbar^{2}k_{l}^{2}}{2M^{⁎}}$$

Using Eq. (27), the following relation is written:

(28) $$E_{\left(k_{l}+q_{l}\right)}^{ex}-E_{\left(k_{l}\right)}^{ex}=\left[E_{g}+\frac{\hbar^{2}}{2M^{⁎}}{\left(k_{l}+q_{l}\right)}^{2}\right]-\left(E_{g}+\frac{\hbar^{2}k_{l}^{2}}{2M^{⁎}}\right)=\frac{\hbar^{2}q_{l}^{2}}{2M^{⁎}}+\frac{\hbar^{2}}{M^{⁎}}k_{l}.q_{l}$$

With

$$E_{g}=\hbar \Omega +2\sum_{i=1}^{N}\sum_{j=1}^{3i}J_{ij}\cos\left(\overset{\to }{k}.\overset{\to }{r_{ij}}\right)$$

By converting the discrete summation into integration as:

(29) $$I_{ex}(k_{l})=\frac{s}{{\left(2\pi \right)}^{2}}\int_{0}^{\infty }dq_{l}\int_{0}^{2\pi }d\theta q_{l}{\left|\chi_{eff}\right|}^{2}\times \left[\frac{1}{\frac{\hbar^{2}q_{l}^{2}}{2M^{⁎}}+\frac{\hbar^{2}}{M^{⁎}}K.q_{l}-\hbar \omega }-\frac{1}{\frac{\hbar^{2}q_{l}^{2}}{2M^{⁎}}+\frac{\hbar^{2}}{M^{⁎}}k_{l}.q_{l}+\hbar \omega }\right]$$

Knowing that:

$$k_{l}.q_{l}=k_{l}q_{l}\cos\theta$$

Eq. (29) becomes:

(30) $$I^{ex}(K)=\frac{S}{{\left(2\pi \right)}^{2}}\int_{0}^{\infty }dq_{l}\int_{0}^{2\pi }q_{l}^{2}d\theta {\left|\chi_{eff}\right|}^{2}\left[\frac{1}{u_{1}+v\cos\theta }-\frac{1}{u_{2}+v\cos\theta }\right]$$

With:

(31) $$u_{1}=\frac{\hbar^{2}q_{l}^{2}}{2M^{⁎}}-\hbar \omega ;\;u_{2}=\frac{\hbar^{2}q_{l}^{2}}{2M^{⁎}}+\hbar \omega ;\;v=\frac{\hbar^{2}}{M^{⁎}}kq_{l}$$

Integrating over *θ* and assuming:

(32) $$T_{1}=\frac{\left(u_{1}-v\right)}{\left(u_{1}+v\right)}=1-\frac{2\hbar^{2}kq_{l}/M^{⁎}}{\frac{\hbar^{2}q_{l}}{M^{⁎}}\left(\frac{q_{l}}{2}+k_{l}\right)-\hbar \omega }$$

and

(33) $$T_{2}=\left(u_{2}+v\right)=\frac{\hbar^{2}q_{l}}{M^{⁎}}\left(\frac{q_{l}}{2}+k_{l}\right)+\hbar \omega$$

Eq. (30) takes the form:

(34) $$I^{ex}(K)=\frac{S}{2\pi }{\left|\chi_{eff}\right|}^{2}\int_{0}^{\infty }dq_{l}q_{l}^{2}\left[\frac{\sqrt{T_{1}}}{\left(u_{1}-v\right)}-\frac{\sqrt{T_{2}}}{T_{2}\sqrt{\left(u_{2}-v\right)}}\right]$$

Assuming k<<q and small exciton wave vectors, expressions of $T_{1}$ and $T_{2}$ in Eq. (34) are approximated as:

(35) $$\sqrt{T_{1}}=\left(1+{\left(\frac{\hbar^{2}k_{l}}{M^{⁎}}\right)}^{2}\frac{q_{l}^{2}}{{\left(\frac{\hbar^{2}q_{l}^{2}}{2M^{⁎}}-\hbar \omega \right)}^{2}}-\left(\frac{\hbar^{2}k_{l}}{M^{⁎}}\right)\frac{q_{l}}{\frac{\hbar^{2}q_{l}^{2}}{2M^{⁎}}-\hbar \omega }\right)$$

And

(36) $$T_{2}\approx \frac{\hbar^{2}q_{l}^{2}}{2M^{⁎}}+\hbar \omega$$

Eqs. (35), (36) into Eq. (34) allow to write:

(37) $$I^{ex}(k_{l})\approx I_{1}^{ex}(k_{l})+I_{2}^{ex}(k_{l})$$

Where

(38) $$I_{1}^{ex}(k_{l})=\frac{S}{2\pi }{\left|\chi_{eff}\right|}^{2}\int_{0}^{\infty }dq_{l}\left[\frac{q_{l}}{\frac{\hbar^{2}q_{l}^{2}}{2M^{⁎}}-\hbar \omega }-\frac{q_{l}}{\frac{\hbar^{2}q_{l}^{2}}{2M^{⁎}}+\hbar \omega }\right]$$

(39) $$I_{2}^{ex}(K)=\frac{S}{2\pi }{\left|\chi_{eff}\right|}^{2}{\left(\frac{\hbar^{2}k_{l}}{M^{⁎}}\right)}^{2}\int_{0}^{\infty }dq_{l}\times \left[\frac{q_{l}^{3}}{{\left(\frac{\hbar^{2}q_{l}^{2}}{2M^{⁎}}-\hbar \omega \right)}^{3}}-\frac{q_{l}^{3}}{{\left(\frac{\hbar^{2}q_{l}^{2}}{2M^{⁎}}+\hbar \omega \right)}^{3}}\right]$$

By performing calculations on Eqs. (38) and (39), we obtain:

(40) $$I_{1}^{ex}(K)=\frac{S}{2\pi }{\left|\chi_{eff}\right|}^{2}\left(\frac{2M^{⁎}}{\hbar^{2}}\right)\left[\frac{1}{2}\ln w\right]$$

(41) $$I_{2}^{ex}(K)=-\frac{S}{2\pi }{\left|\chi_{eff}\right|}^{2}{\left(\frac{\hbar^{2}k_{l}}{M^{⁎}}\right)}^{2}{\left(\frac{2M^{⁎}}{\hbar^{2}}\right)}^{3}\left(\frac{1}{2w}\right)$$

With $w=\frac{2M^{⁎}\omega }{\hbar }$

(42) $$I_{2}^{ex}(k_{l})={\left(\frac{\hbar^{2}k_{l}^{2}}{2M^{⁎}}\right)}^{2}I_{3}^{ex}(k_{l})$$

With

(43) $$I_{3}^{ex}(k_{l})=-\frac{S}{\pi }{\left|\chi_{eff}\right|}^{2}\frac{8M^{⁎2}\omega }{\hbar^{5}}$$

The ground state energy becomes:

(44) $$E_{pol}^{ex}(0)=\left(\frac{\hbar^{2}k_{l}^{2}}{2M^{⁎}}\right)\left(1+{\overset{˜}{I}}_{3}^{ex}\right)+E_{g}+E_{z}^{ex}+\hbar \Omega +\hbar \omega -I_{1}^{ex}(k_{l})$$

### 2.4 Mobility of exciton-polaron in MTs

The mobility of charge carriers is a well-known concept applied in solid-state physics to characterize the speed at which an electron can move through a material when it is influenced by an external phenomenon. Mobility will allow us to characterize the movement of quasi-particles in microtubules. Based on quantum statistical theory (Sun et al., 2014; Coehoorn et al., 2017; Fobasso et al., 2020), the average number of phonons is given by:

(45) $$\bar{N}=\frac{1}{2}{\left[\exp\left(\frac{E_{0}}{K_{B}T}\right)-1\right]}^{-1}$$

Where *T* and $K_{B}$ are respectively the temperature and Boltzmann constant. Where $E_{0}$ is the fundamental state energy. The exciton-polaron mobility *μ* (Fobasso et al., 2020) will be given by the following formula:

(46) $$\mu \approx \frac{1}{\bar{N}}=2\left[\exp\left(\frac{E_{0}}{K_{B}T}\right)-1\right]$$

### 2.5 Tsallis entropy

Tsallis entropy is a measure of disorder at a local point in the system. It is linked to Gibbs and Boltzmann entropy by the parameter *x* (Tsallis, 1995; Kenfack-Sadem et al., 2020). The Tsallis entropy (Tsallis, 1988) is a generalization of the Boltzmann-Gibbs entropy (Havrda and Charvát, 1967) corresponding to the quantity of information contained or delivered by an information source. In the microtubule system, Tsallis entropy quantifies the amount of information that the exciton-polarons exchange with its environment.

(47) $$S_{x}=k\frac{1-\sum_{i}^{n}P_{i}^{x}}{x-1}$$

Where *k* is the conventional constant, and *x* is any real number, $P_{i}$ is the probability of microscopic configuration and *n* is the number of the microscopic configuration of the system. For x→1, the connection to thermodynamics is established as (Kenfack-Sadem et al., 2020).

(48) $$\frac{1}{T}=\frac{\partial S_{x}}{\partial n_{x}}$$

Where $T=\frac{1}{\beta K_{B}}$

The free energy is given by:

(49) $$F_{x}\equiv U_{x}-TS_{x}=-\frac{1}{\beta }\ln Z_{x}$$

With

(50) $$U_{x}=-\frac{\partial }{\partial \beta }\ln Z_{x}$$

For

(51) $$x\to 1S_{x}=\ln Z_{x}+\beta U_{x}$$

It follows that:

$$P_{i}^{x}=\left\{ \begin{array}{c} \frac{{\left[1-\beta \left(1-x\right)E_{i}\right]}^{\frac{1}{1-x}}}{Z_{x}}\\ 0\;\text{otherwise} \end{array}\right.\;\mathrm{If}1-\beta \left(1-x\right)E_{i}≻0$$

Where the partition function is given by:

(52) $$Z_{x}={\left[1-\beta \left(1-x\right)E_{ex}^{pol}\right]}^{\frac{1}{1-x}}$$

With $E_{i}=E_{ex}^{pol}$ the energy of the corresponding microstate and:

(53) $$U_{x}=\frac{E_{ex}^{pol}}{1-\beta \left(1-x\right)E_{ex}^{pol}}$$

The final expression of the Tsallis entropy is given by:

(54) $$S_{x}=\left(\frac{1}{1-x}\right)\ln\left[1-\beta \left(1-x\right)E_{ex}^{pol}\right]+\beta \left[\frac{E_{ex}^{pol}}{1-\beta \left(1-x\right)E_{ex}^{pol}}\right]$$

## 3 Numerical results

**Figure 2:**
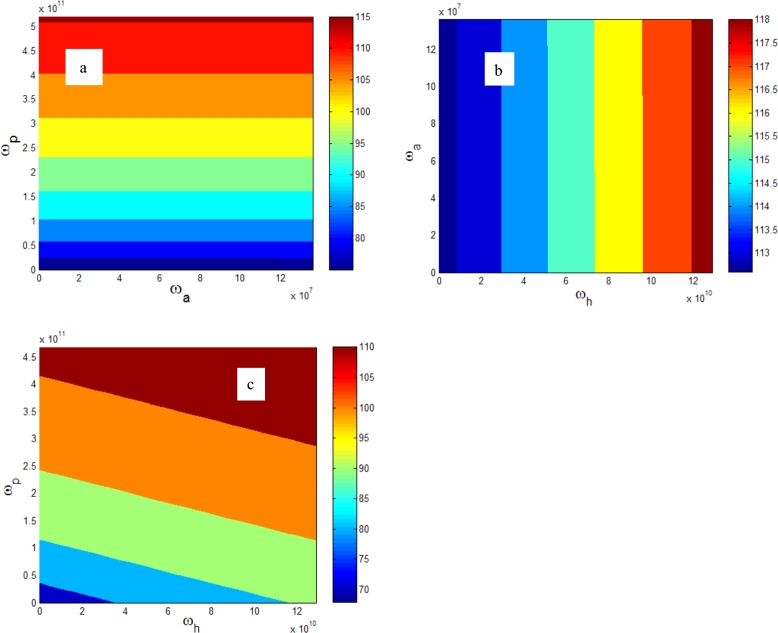
a): exciton-polaron energy as a function of vibration frequency along the main helix *ω*_*h*_ and the vibration frequency along the antihelix *ω*_*a*_. b): exciton-polaron energy as a function of vibration frequency along the antihelix and the vibration frequency along a protofilament *ω*_*p*_. c): exciton-polaron energy as a function of the vibration frequency along the main helix *ω*_*h*_ and the vibration frequency along a protofilament *ω*_*a*_.

In this section to properly observe the exciton-polaron dynamics in microtubules, numerical simulations of the energy, mobility, and entropy of the ground state are performed using the MATLAB simulation software. To obtain the different frequencies, we varied the elasticity constants: the elasticity constant along the protofilament $k_{p}$ between 0 and 5 N/m, the elasticity constant along the helix $k_{h}$ between 0 and 1 N/m, and the elasticity constant along the antihelix $k_{a}$ between 0 and 0.1 N/m. The MT's parameters are selected efficiently in order to highlight the effect of vibrations on the properties of the exciton-polaron. The vibration frequencies taken in Fig. 2 are in the range of the vibration frequencies obtained by (Portet et al., 2005; Sirenko et al., 1996a, Sirenko et al., 1996b). We have plotted (Fig. 2) the energy of the exciton-polaron as a function of the frequencies of vibrations following the protofilament, the helix, and the antihelix.

In Fig. 2a, we observe that the exciton-polaron energy is constant according to the antihelix vibrations but varies with the protofilament's vibration. In Fig. 2b, we observe that the exciton-polaron energy is constant according to the antihelix vibrations but varies with the helix vibrations. In Fig. 2c, we observe that exciton-polaron energy varies according to the vibrations of both protofilament and helix and this energy is higher with the vibrations of the protofilament. We claim that the energy of the exciton-polaron is anisotropic and sensitive to vibrations following the protofilament and the helix but is not sensitive to vibrations following the antihelix. This energy is stronger in the protofilament than in the helix. It is reported in the work of Portet et al. (2005) that the vibrations which propagate along the protofilament do so much faster than those along the helix and that the frequency of vibration of the protofilament is greater than the frequency of vibration of the helix. We can conclude that the energy of exciton -polaron is anisotropic and increases with phonon velocity. We can also say that the electron, hole, and phonon couplings are higher in the protofilament than in the helix. Exciton-polaron is more spread and more dynamic in the protofilament than the helix. Mobility can support this statement.

**Figure 3:**
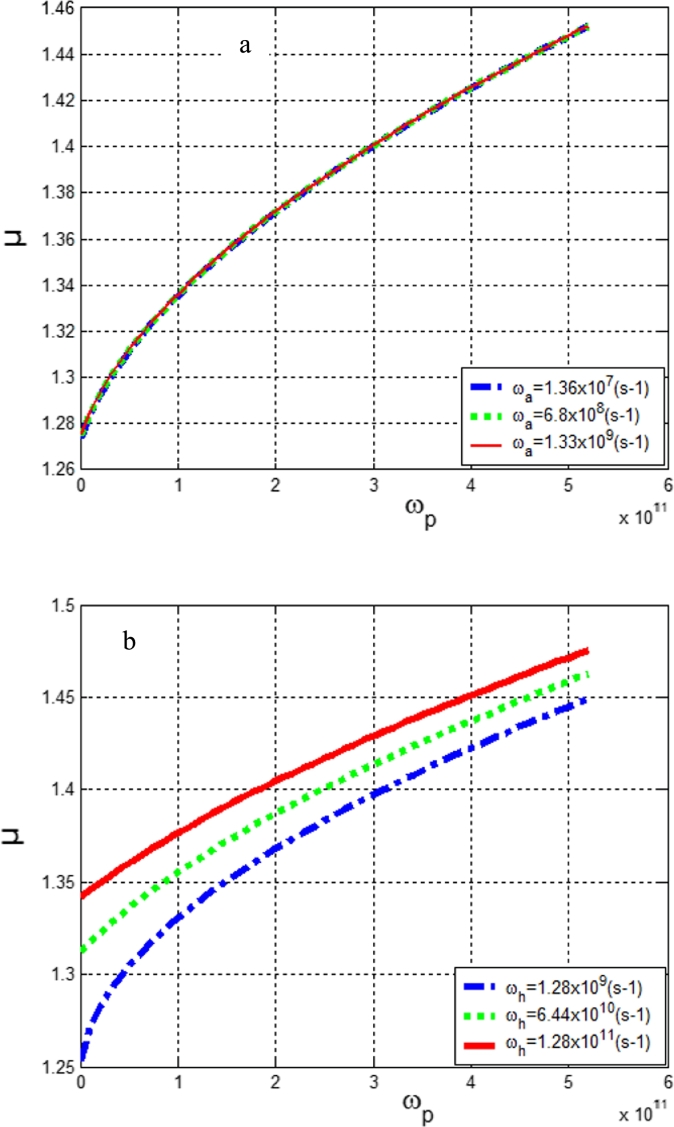
a): Mobility *μ* as a function of vibration frequency along a protofilament for different values of the helix frequency *ω*_*h*_. b): Mobility as a function of vibration frequency along a protofilament for different values of the antihelix frequency *ω*_*a*_.

Fig. 3 presents the mobility of charge carriers as a function of the vibrations along the protofilament for different values of the vibrations along the helix and antihelix respectively. Fig. 3a shows that the mobility increases with the vibrations along the protofilament and remains constant for different vibrations along the antihelix. In Fig. 3b, the mobility increases with the vibrations of the protofilament and the helix. This result shows how the charge carriers move in the microtubules under the action of an anisotropic electric field created by the vibrations along the helix and the protofilament but, absent in the antihelix. These results are in agreement with those reported in the work of Pokorný et al. (2005) and confirm that the transport of charge carriers in microtubules is anisotropic and well depending on the protofilament than the helix. From Fig. 3a, we can suggest that the antihelix vibrations do not produce an intrinsic electric field (Nganfo et al., 2021) contributing to the self-organization of microtubules. This exciton-polaron mobility leads to the exchange of information between the quasiparticle and its environment (Chang et al., 2006; Kenfack-Sadem et al., 2021). So it will be important to evaluate the entropy of the quasi-particle in the system.

**Figure 4:**
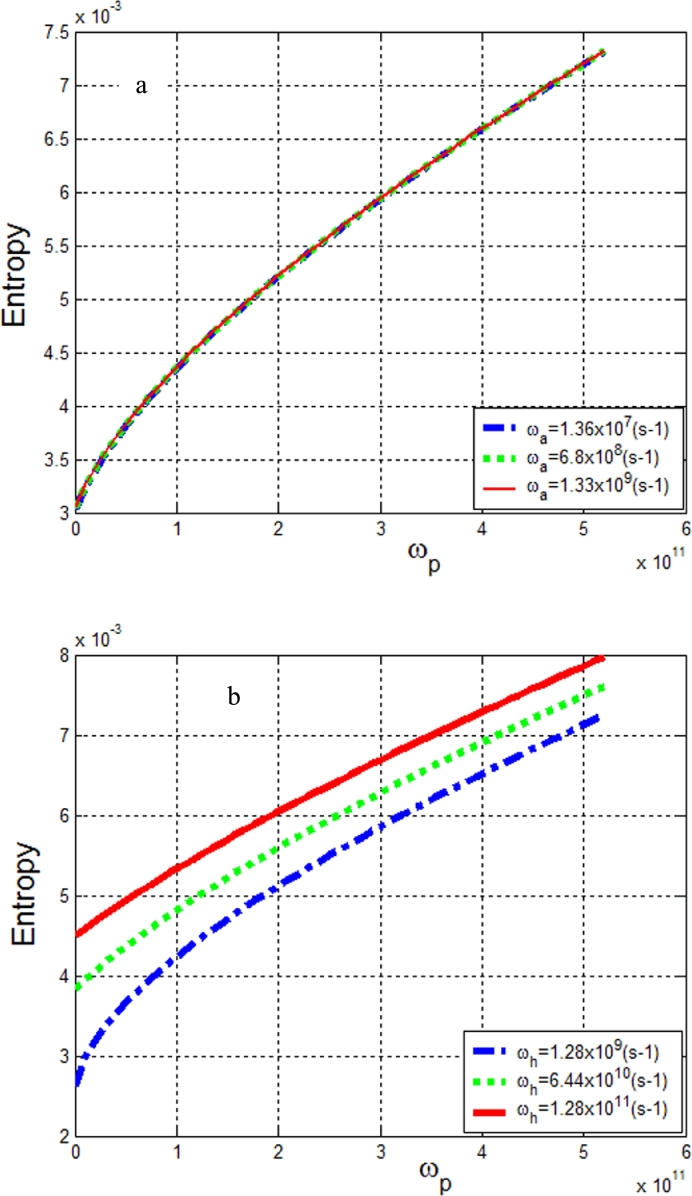
a): Tsallis entropy as a function of vibration frequency along a protofilament *ω*_*p*_ for different values of the helix frequency *ω*_*h*_. b): Entropy as a function of vibration frequency along a protofilament for different values of the antihelix frequency *ω*_*a*_.

In Fig. 4, we evaluated the Tsallis entropy as a function of the vibrations along the protofilament for different values of the vibrations along the helix and the antihelix. In Fig. 4a, we observe that the entropy increases with the protofilament vibrations and remains constant for different antihelix vibrations. In Fig. 4b, the entropy increases with the vibrations along the protofilament and the helix. This can lead to the fact that MT structures possess strong robustness to static disorder in comparison to geometries that include only short-range interactions. These results suggest that the dynamics of the exciton-polaron in MTs occurs with the exchange of information with its environment. This information is higher at the protofilament than at the helix.

**Figure 5:**
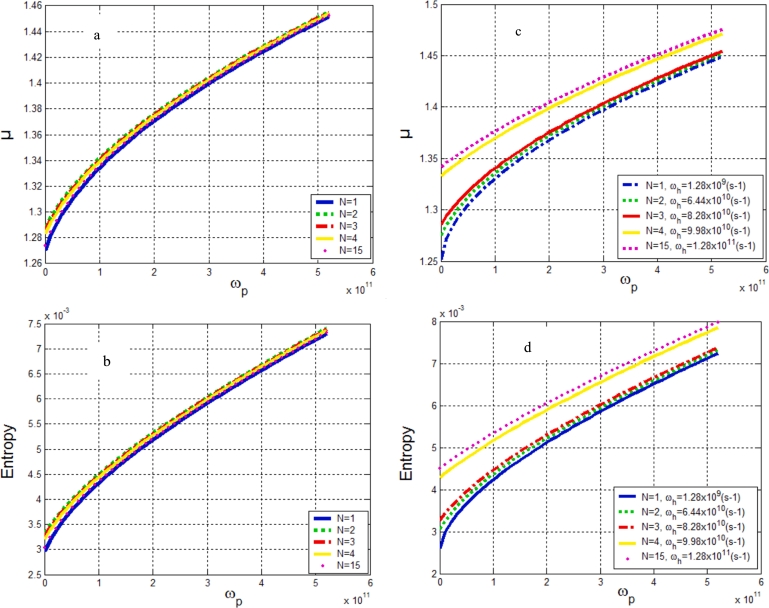
a): Mobility *μ* as a function of vibration frequency along a protofilament *ω*_*p*_ for the population of protofilament. b): Entropy as a function of vibration frequency along a protofilament for the population of protofilament. c): Mobility *μ* as a function of vibration frequency along a protofilament for the population of protofilament and for different values of the helix. d): Entropy as a function of vibration frequency along a protofilament *ω*_*p*_ for the population of protofilament and for different values of the helix *ω*_*h*_.

Figs. 5a and 5b show the behavior of the entropy and the mobility. When the vibrations along the helix are set at 0.1 s^−1^, the entropy and the mobility increase from 1 to 2 protofilaments. From 3 protofilaments up to 15, the entropy and the mobility decrease with the vibrations along the protofilament. In Figs. 5c and 5d, it is observed that when the helix vibrations are no longer fixed, the entropy and the mobility increase for 1 to 15 protofilaments. This result confirms the importance of helix vibrations which are neglected by many heights (Cifra et al., 2011; Thackston et al., 2019). When the intensity of vibration of the helix is fixed, the behavior of the exciton-polaron is identical to the process of information exchange in the system. We find that the mobility of the exciton-polaron is increasing between the first and second protofilament. From the third protofilament until the last this mobility decreases. When the vibrations of the helix and protofilament vary, the mobility of exciton-polaron and entropy is increasing from the first to the last protofilament. The protofilament and the helix vibrations affect the mobility and the entropy of the system. Results obtained in this subsection are similar to those obtained in refs (Fotue et al., 2021; Pokorný et al., 2005). Therefore the dynamic of exciton polaron is improved when the number of protofilament increases. In addition, the latter promotes the exchange of information between the quasi-particle and its environment. This can be a good way to restore the stability of MT.

## 4 Conclusions

We investigated the dynamical properties of the exciton-polaron in the microtubule. The study was carried out using a unitary transformation and an approximate diagonalization technique. Analytically, the expressions of the ground-state energy, mobility, and the Tsallis entropy of the exciton-polaron have been derived according to the parameters characterizing the geometry of the microtubules. The numerical results based on parameters of microtubules show that the energy of exciton-polaron is anisotropic and increases with phonon velocity. The vibration frequencies chosen are in the range of the vibration frequencies obtained in the literature. We found that the energy of the exciton-polaron is anisotropic and sensitive to vibrations through the protofilament and the helix but it is not the case through the antihelix. This energy is stronger in the protofilament than in the helix. Exciton-polaron is more spread and more dynamic in the protofilament than the helix. The mobility of the exciton-polaron is not sensitive to the vibration along the antihelix. The dynamics of exciton-polaron in MTs vary with the exchange of information with its environment as a function of the vibrations of helix and protofilament. This information is higher at the protofilament level than the helix. When we fix the vibration values of the helix, the behavior of the exciton-polaron is identical to the process of information exchange in the system. We find that the mobility of the exciton-polaron is increasing between the first and second protofilament. From the third protofilament until the last this mobility decreases. This can be interesting in order to stabilize MT. When the vibrations of the helix and protofilament vary, the mobility of exciton-polaron and entropy is increasing from the first to the last protofilament. This work can be interesting to solve the problem in cellular organization and knowledge processing. At the end of this work, we found that the role of exciton-polaron in the dynamic instability of microtubules is an important aspect that is important to investigate.
